# Enhanced Triboelectric Performance of Modified PDMS Nanocomposite Multilayered Nanogenerators

**DOI:** 10.3390/ma13184156

**Published:** 2020-09-18

**Authors:** Habtamu Gebeyehu Menge, Jin Ok Kim, Yong Tae Park

**Affiliations:** Department of Mechanical Engineering, Myongji University, 116 Myongji-ro, Cheoin-gu, Yongin, Gyeonggi 17058, Korea; chengdep@gmail.com (H.G.M.); 10gncn@naver.com (J.O.K.)

**Keywords:** triboelectric nanogenerator, layer-by-layer assembly, roughness, charge density, graphene

## Abstract

Recently, triboelectric nanogenerators (TENGs) have been widely utilized to address the energy demand of portable electronic devices by harvesting electrical energy from human activities or immediate surroundings. To increase the surface charge and surface area of negative TENGs, previous studies suggested several approaches such as micro-patterned arrays, porous structures, multilayer alignment, ion injections, ground systems and mixing of high dielectric constant materials. However, the preparation processes of these nanocomposite TENGs have been found to be complex and expensive. In this work, we report a simple, efficient and inexpensive modification of poly(dimethylsiloxane) (PDMS) using graphene nanoplatelets (GNPs) fillers and a Na_2_CO_3_ template. This GNP-PDMS was chemically bonded using 3-aminopropylethoxysilane (APTES) as a linker with an electrode multilayer made by layer-by-layer deposition of polyvinyl alcohol (PVA) and poly(4-styrene-sulfonic acid) (PSS)-stabilized GNP (denoted as [PVA/GNP-PSS]*_n_*). A 33 wt.% Na_2_CO_3_ and 0.5 wt.% of GNP into a PDMS-based TENG gives an open-circuit voltage and short-circuit current density of up to ~270.2 V and ~0.44 μA/cm^2^, which are ~8.7 and ~3.5 times higher than those of the pristine PDMS, respectively. The higher output performance is due to (1) the improved surface charge density, 54.49 μC/m^2^, from oxygen functional moieties of GNP, (2) high surface roughness of the composite film, ~0.399 μm, which also increased the effective contact area, and (3) reduced charge leakage from chemical bonding of GNP-PDMS and [PVA/GNP-PSS]_3_ via APTES. The proposed TENG fabrication process could be useful for the development of other high-performance TENGs.

## 1. Introduction

Triboelectric nanogenerators (TENGs) are an emerging technology for scavenging renewable mechanical energy from the environment by the coupled effect of triboelectrification and electrostatic induction. The electricity generation mechanism of TENGs is based on triboelectrification and electric induction due to the periodical contact and separation movements of two tribomaterials (TMs) that have different triboelectric coefficients. For good electric output performance, TMs are coupled based on their large difference in electron attracting ability from the triboelectric series [1,2,3,4]. There are four fundamental operation modes of the TENG: vertical contact separation mode, in-plane sliding mode, single electrode mode and freestanding triboelectric layer mode [5]. Among these TENG modes, the vertical contact separation mode was first invented and considered as the principal mode of TENG due to low cost, simple design, device stability, prolonged life span and high electric output performance [6].

Material selection is one of the basic parameters to fabricate TENG units for stable and better performance. Even though there are plenty of TMs for TENG fabrication [1,7,8,9], poly(dimethylsiloxane) (PDMS) has been extensively studied as a negative TM [10,11,12,13]. This is due to the fact that PDMS is a highly electronegative, hydrophobic, flexible, cost effective, optically clear, inert and non-toxic TM and has the potential to make composite films by mixing with nanoparticles (NPs) [14] and creating a microstructure [15].

Among the TENG control parameters, capacitance and surface area are two important parameters that significantly change the performance of the TENG [3]. For this reason, the use of nanofillers or NPs increases the capacitance of TENGs, resulting in a higher surface charge density of the PDMS polymer [10,11,12,13]. However, if these nanoparticles exceed their optimum values, the dielectric properties of PDMS may decrease and ultimately the output voltage may decrease. Therefore, it is important to determine the optimal value of the nanoparticles in PDMS [13]. On the other hand, when the microstructure is introduced as a sacrificial template into the PDMS film, the surface area is increased and the total amount of charge generated after contact increases [7,10,15,16,17]. The introduction of the sacrificial template also improves the hydrophobicity of PDMS, making the TENG’s performance less sensitive to humidity [15]. PDMS modifications have been investigated to achieve high electrical output performance in PDMS-based TENGs; some of them are PDMS/carbon nanotube (CNT)/NaCl [7], etched PDMS/CNT [8], PDMS/sugar [10], PDMS/graphite NPs [13], PDMS/SrTiO_3_/NaCl [16], PDMS/Na_2_CO_3_ [15], PDMS/nature replicated layer [18], PDMS/BaTiO_3_ NPs [19], PDMS/monolayer [20] and PDMS/aligned graphene sheet [21]. They have exhibited enhanced electrical output performance compared to pristine PDMS films. Meanwhile, PDMS mixed with graphite NPs [13], PDMS/aligned graphene sheet [21] and Na_2_CO_3_ [15] showed higher electrical output performance of the TENG. These previous studies, however, differ from this one, which investigated the coupling effect of the two parameters (i.e., simultaneous increase in capacitance and surface area of PDMS films) that improves the electrical output performance of the TENG using both GNP and Na_2_CO_3_. Regarding the other aspect of the process, various manufacturing techniques have been used to improve the capacitance and surface area of the PDMS film such as heating [22], plasma treatment [8,23], ion doping [24], soft lithography [18], micro-patterned arrays [25], porous structures and multilayer alignment [21,26] and mixing with high dielectric constant materials [16]. Most of the aforementioned manufacturing techniques are complex, energy intensive and low reproducibility processes. Therefore, in order to overcome these shortcomings, it is necessary to use an effective modification method for PDMS films through a simple, inexpensive and eco-friendly process.

Recently, it has been discovered and reported that the loss of electrically induced positive charge gradually reduces the TENG performance [27,28,29]. To the best of our knowledge, the loss of electrically induced positive charge blocked by the physical adhesion between the negative TM and electrode has not been highlighted, thus the chemically bonded interface should be considered.

In this study, we reported a facile and simple fabrication procedure of PDMS-based TENGs that affects both capacitance and surface area. To be specific, the structurally modified PDMS was prepared by employing GNP as a conductive nanofiller and Na_2_CO_3_ salt as a sacrificial template. We also proposed a chemically bonded interface between the negative TM and the electrode to improve the electrical output performance of the GNP-PDMS system. For this purpose, we incorporated a GNP-based polymer composite electrode made of polyvinyl alcohol (PVA) and poly(4-styrene-sulfonic acid) (PSS)-stabilized GNP, denoted as [PVA/GNP-PSS]*_n_*. These [PVA/GNP-PSS]_3_ multilayers were prepared using an inexpensive and simple layer-by-layer (LbL) assembly process, which was advantageous for precise control of the thickness of the film [4,30,31,32]. Then, the modified PDMS negative TM layer and [PVA/GNP-PSS]_3_ multilayer electrode were chemically linked by 3-aminopropylethoxysilane (APTES) as a coupling agent under mild reaction conditions without altering the desired properties of the composite films [33,34]. Finally, the GNP-PDMS nanocomposites, fabricated by adding 33 wt.% Na_2_CO_3_ and 0.5 wt.% GNP and assembled with a [PVA/GNP-PSS]_3_ electrode, achieved an open-circuit voltage and short-circuit current density of up to 270.2 V and 0.44 μA/cm^2^, respectively. This modified PDMS-based TENG fabrication process could be useful for the development of other high-performance TENGs.

## 2. Materials and Methods

### 2.1. Materials

PDMS (Silgard 184A), PSS solution (18 wt.% in H_2_O), APTES, toluene (anhydrous, 99.8%), PVA powder and Na_2_CO_3_ powder (anhydrous, ≥99.5%) were bought from Sigma-Aldrich (Seoul, Korea) Poly terephthalate (PET) film was bought from Goodfellow (Seoul, Korea). GNP (N002-PDR, X-Y dimensions of 10 mm at most, carbon content ≥95%, oxygen content ≤2.5%) was provided by Angstron (Dayton, OH, USA).

### 2.2. Fabrication of Modified PDMS Composite Films

**Figure 1:**
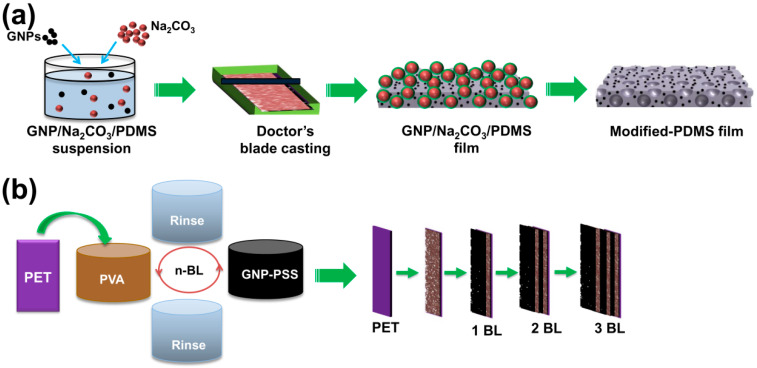

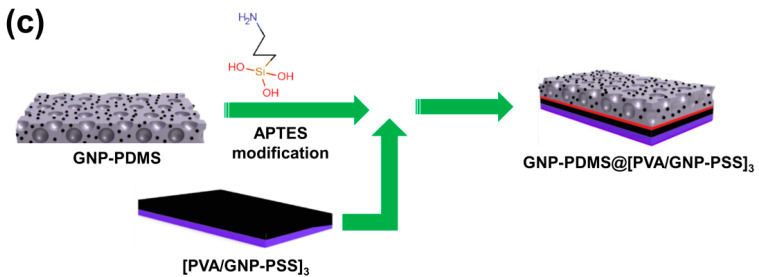
Fabrication procedure of the GNP-PDMS@[PVA/GNP-PSS]_3_ TENGs. (**a**) GNP-PDMS preparation process. (**b**) LbL assembly of [PVA/GNP-PSS]_3_ multilayers. (**c**) APTES-mediated linkage of GNP-PDMS and [PVA/GNP-PSS]_3_ composite films.

Figure 1a shows the schematic diagram of the rough surface GNP-PDMS composite film fabricating process. In this process, the PDMS solution was comprised of both an elastomer and a curing agent in a mass ratio of 10:1. First, 0.03 and 0.50 wt.% GNPs were dispersed in toluene, respectively, and then mixed with elastomer by bath sonicating for 30 min. The solution was then heated at 65 °C for 2 h to completely evaporate the toluene. Second, the curing agent was added into the well-homogenized solution with bath sonication for 15 min to obtain a uniformly mixed dispersion. Third, 33 wt.% of Na_2_CO_3_ salt was put into the well-mixed GNP-PDMS dispersions, and mixed by a vortex mixer for 30 min followed by degassing using a vacuum desiccator for 20 min. Fourth, the dispersions were cast into film shapes by spreading on a mold using the doctor’s blade casting technique. Subsequently, the samples were kept at room temperature for 45 min in a vacuum desiccator and then thermally cured in a pre-heated oven at 80 °C for 45 min. Finally, after cooling to room temperature, the cured composite films were peeled off gently from the mold and dipped in deionized (DI) water for 12 h under bath sonication to completely remove the Na_2_CO_3_ particles. The modified GNP-PDMS composite films were obtained after washing with DI water and drying in a vacuum oven at 60 °C for 30 min. The GNP-PDMS composite films obtained using 0.03 and 0.50 wt.% GNP were denoted as SG-PDMS-I and SG-PDMS-II, respectively. Pristine PDMS film and PDMS film prepared by adding 33 wt.% Na_2_CO_3_ salt without the GNP fillers (hereafter, S-PDMS) were also fabricated using a similar procedure.

### 2.3. Fabrication of [PVA/GNP-PSS]_n_ Film by LbL Assembly

The LbL assembly of [PVA/GNP-PSS]*_n_* film was performed according to the procedure described elsewhere [30]. PVA solution (0.25 wt.%) was prepared by dissolving PVA powder in DI water. PSS-stabilized GNP aqueous suspension (henceforth, GNP-PSS suspension) containing 0.1 wt.% GNP and 0.1 wt.% PSS was prepared by co-mixing GNP nanoplatelets and PSS in DI water and then homogenized for 3 h using a tip sonicator (Sonoplus HD-1070, Bandelin, Berlin, Germany). The multilayer coating of the [PVA/GNP-PSS]*_n_* film was carried out by depositing on a 2.2 cm × 2.2 cm × 100 μm PET substrate. First, the substrate was cleaned by sonicating in IPA solution for 5 min using a bath sonicator followed by oxygen plasma treatment (PDC-32 G-2, Harrick Plasma, Ithaca, NY, USA) for 5 min to promote the conformal deposition of the first PVA layer. Then, a protective film was coated on one side of the PET surface. Finally, the substrate was alternatively dipped in a PVA solution and GNP-PSS dispersion. The dipping duration in each solution was 5 min. Additionally, every dipping process was followed by a cleaning process consisting of washing with DI water twice and air drying. One cycle was one bilayer (1 BL), and the process was halted after deposition of the desired number of BLs as shown in Figure 1b.

### 2.4. Fabrication of GNP-PDMS@[PVA/GNP-PSS]_n_ Composite TENGs

GNP-PDMS@[PVA/GNP-PSS]*_n_* composite TENGs were prepared using APTES as a linker (Figure 1c). First, the PDMS films fabricated above were modified with APTES to bear amine functionality according to the method described elsewhere [33] at mild reaction conditions without alteration of the desired properties of the composite films. Briefly, each PDMS composite film was immersed in APTES:ethanol solution (1:2 *v*/*v*) and left at room temperature for 5 min under vigorous stirring for complete hydrolysis as well as diffusion of the hydrolysed APTES solution on the rough surface of the GNP-PDMS composites. Subsequently, after drying for 1 min, the films were dipped into aqueous acetic acid solution (33% *w*/*v*) for 3 min to promote condensation of APTES on the surface of the PDMS films by forming a siloxane network. The samples were then dried overnight under ambient conditions. Finally, each APTES-modified PDMS film was attached on the PSS side of the [PVA/GNP-PSS]*_n_* composite film due to the electrostatic attraction between the amino groups in the PDMS films and sulfonate group of PSS, giving multilayered TENG composites shown in Figure 1c after drying overnight at room temperature.

### 2.5. Characterization

The surface morphology of the composite films was characterized with a scanning electron microscope (SEM, EM-30AX, COXEM, Daejeon, Korea) operated at 20.0 KV. Light transmittance of the samples was examined by measuring the absorbance at 500 nm using a UV-visible spectrometer (DH-2000-BAL, Oceans Optics, Orlando, FL, USA). To determine the hydrophobicity of the samples, water contact angle measurements were performed using a static angle measurement instrument (SDS-TEZD10012, Femtofab, Seongnam, Korea) using about 4.8 μL water droplets at 27.4 °C. Sheet resistance measurement was carried out using a four-point probe with a 0.4 mm probe tip diameter and 1.0 mm tip spacing (Pro4, Signatone, Gilroy, CA, USA), and power was supplied using an E3644A DC (Agilent Technologies, Santa Clara, CA, USA) with an operating voltage of 10 V and a digital multimeter (2001, Keithley Instruments, Cleveland, OH, USA). The surface roughness was measured using an atomic force microscope (AFM, UNHT³, Anton Paar, Graz, Austria).

### 2.6. Electrical Output Performance Measurement

The building blocks of the fabricated TENGs are [PVA/GNP-PSS]_3_ LbL-assembled thin film, modified PDMS, PET and aluminium (Al). PDMS and PET were chosen as negative and positive TMs, respectively. Al attached on PET by adhesive tape and the [PVA/GNP-PSS]_3_ composite film were utilized as top and bottom electrodes, respectively. Two copper wires were connected to both electrodes for electric measurement. The dimensions of the TENGs were 2.2 cm × 2.2 cm × 400 μm with a 10 mm gap between the top electrode and the triboelectric PDMS layer. The electrical output performance of the TENGs was evaluated using a vertical contact separation mode. Contact frequency and compressive forces were kept constant at 4 Hz and 10 N, respectively, for all samples. Electrical outputs, open-circuit voltage (V_OC_) and short-circuit current (I_SC_) were measured using an oscilloscope (MDO3052, Tektronix, Beaverton, OR, USA), a low noise current preamplifier (SR570, Stanford Research Systems) and an electrometer (6514 system electrometer, Keithley, Cleveland, OH, USA).

## 3. Results and Discussion

### 3.1. Characterization of Composite Films

**Figure 2:**
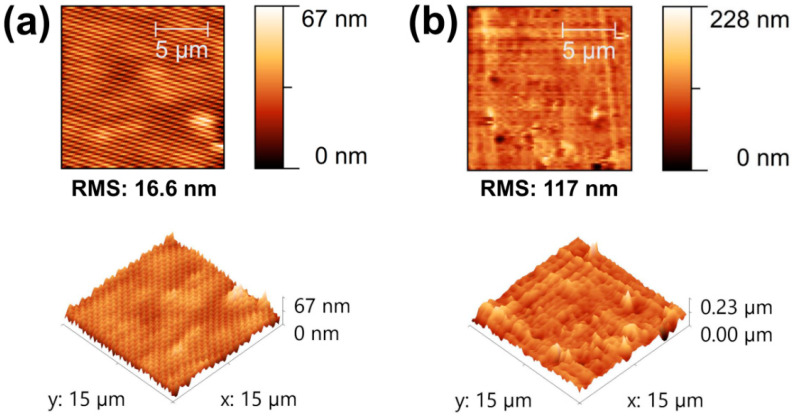

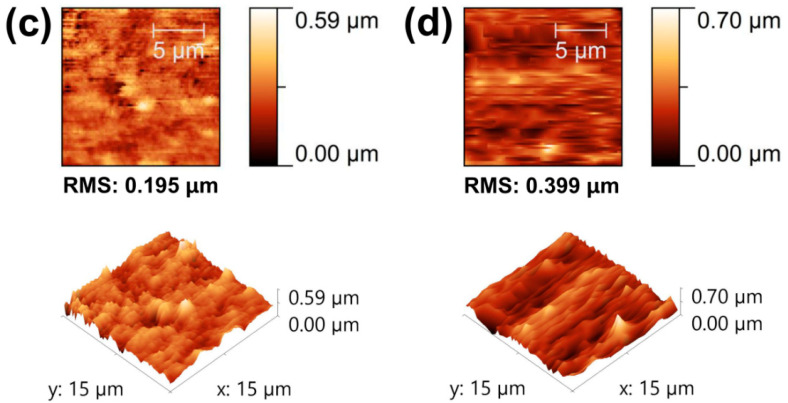
AFM 2D and 3D images (**a**–**d**) of PDMS, S-PDMS, SG-PDMS-I and SG-PDMS-II of composite films, respectively.

Generally, the output performance was increased by the enhanced effective contact area, compressive stress and surface charge density. Figure 2 shows the two- and three-dimensional AFM surface images of each modified PDMS sample with the RMS roughness for closer examination. The observed roughness values were 16.6 nm, 117 nm, 0.195 μm and 0.399 μm for PDMS, S-PDMS, SG-PDMS-I and SG-PDMS-II, respectively, which agreed with their top and cross-sectional SEM images (Figures S1 and S2, respectively). These surface roughness increases were caused by the pores by the Na_2_CO_3_ sacrificial template and nanoparticles by GNP loading onto the PDMS layer.

Theoretically, as shown in Figure S3, pristine PDMS has a smooth surface compared to other PDMS composite films. On the contrary, S-GPDMS showed a porous and rough surface due to the addition of the Na_2_CO_3_ template. The porosity would be similar for SG-PDMS-I and SG-PDMS-II along with the loading of GNP. Both SG-PDMS-I and SG-PDMS-II had the same Na_2_CO_3_ concentration. However, they exhibited different morphology due to the addition of a large amount of GNP for SG-PDMS-II, showing more GNP distribution on the inner wall of the pores as well as the outer surface. These theoretical predictions were supported by SEM images (Figures S1 and S2) and AFM analysis (Figure 2).

To demonstrate these coupling effects of the loading of GNP and the Na_2_CO_3_ sacrificial template, we compared the charge density of PDMS, SG-PDMS-I and SG-PDMS-II TENGs. As shown in Figure S4, remarkably, the surface charge density of SG-PDMS-II/TENG reached 54.49 μC/m^2^, which is 2.5 and 1.6 times higher than pristine PDMS/TENG and SG-PDMS-I/TENG, 22 and 32.4 μC/m^2^, respectively.

**Figure 3:**
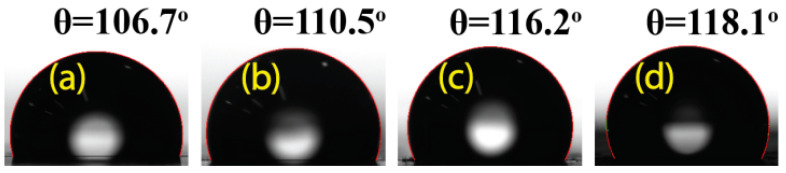
Water contact angles of water droplets on each sample. (**a**) PDMS; (**b**) S-PDMS; (**c**) SG-PDMS-I and (**d**) SG-PDMS-II.

Hydrophobicity was used as evidence of functional implementation of GNP inside the PDMS layer. The hydrophobicity of each composite material was examined by measuring the contact angle (θ_CA_) under the humid air (RH ~42%) condition. The standard deviation of the water contact angle was found to be approximately ± 0.5° and a very small droplet of water ~4.8 μL was used at 27.4 °C for each sample. As shown in Figure 3, θ_CA_ increased from PDMS to S-PDMS and SG-PDMS, sequentially, showing more hydrophobic surface due to the greater roughness and higher hydrophobic GNP loading. It has been known that the surface morphology influences the change in hydrophobicity as mentioned elsewhere [15].

Furthermore, this improvement in hydrophobicity is one of the criteria for choosing the best TENG devices. For the operation of TENGs in a humid environment, it is necessary to have a character of hydrophobicity along with other TENG properties. All samples exhibited a hydrophobic surface with θ_CA_ greater than 90°, and the highest value, θ_CA_ = 118.1°, was obtained for the SG-PDMS-II film, which contained 0.5 wt.% of GNP. This hydrophobic surface allowed the TENG material to operate in humid environmental conditions.

**Figure 4:**
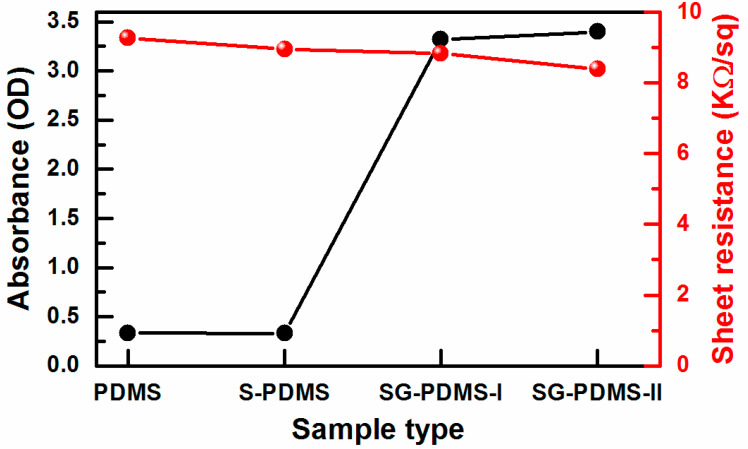
Absorbance (at 500 nm) and sheet resistance of the GNP-PDMS@[PVA/GNP-PSS]*_3_* composite TENGs. For simplicity, only the GNP-PDMS sample labels are displayed in the x-axis.

The transparency of the materials was examined by measuring the absorbance at 500 nm using UV-Vis spectroscopy (Figure 4). The absorbance of PDMS remained unchanged after the structural modification by Na_2_CO_3_ salt, which was around 0.34 optical density (OD) for both PDMS and S-PDMS. However, the absorbance was significantly increased to approximately 3.40 OD for SG-PDMS-II, due to the presence of optically opaque GNPs.

The dielectric property of a TM is vital for obtaining high electric output performance. To check whether the dielectric property of PDMS was affected after surface modification or not, the sheet resistance of the films was measured. The resistivity of the composite films decreased slightly as the GNP loading increased (Figure 4). Nonetheless, this change was not significant enough to alter the dielectric property of the PDMS-based composite films. Thus, their electrical output performance and mechanical durability were subsequently investigated.

### 3.2. Electrical Output Performance of TENGs

**Figure 5:**
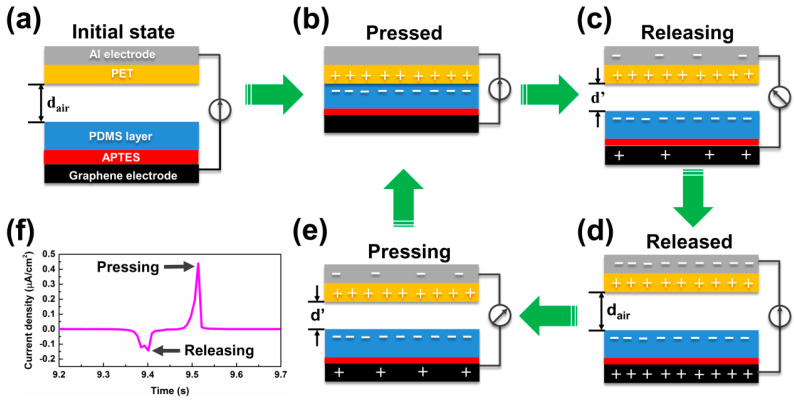
(**a**) Theoretical model, and (**b**–**e**) proposed charge generation mechanism of the developed TENG. (**f**) One cycle of the generated current output signal.

The electricity generation mechanism of the TENGs with a vertical contact separation mode is briefly depicted in Figure 5 and a finite element simulation of the potential distribution in the SG-PDMS-II/TENG was illustrated using COMSOL Multiphysics^®^ (Ver.5.2, Altsoft, Seoul, Korea) (Figure S5). In the absence of compressive force, the TM surfaces were not in contact and thus there was no charge transfer (Figure 5a). When the two TMs were brought into contact with an external pushing force (Figure 5b), charges were generated at the interface of the two TMs. At this point, the negative triboelectric charges accumulated on the PDMS layer due to its strong electronegativity, while positive charges appeared on the PET surface. When the compressive force was released (Figure 5c), the balance of triboelectric potential was upset. As a result, an electrostatically induced charge flowed between the electrodes through an external load, which created a current flow until the device was fully restored to its original state, as shown in Figure 5d. Once an external force was applied again, a potential difference was created again due to the reduced interlayer gap distance, which resulted in an opposite current flow, as illustrated in Figure 5e. In the course of the periodical pressing and releasing processes, an alternating output signal was generated in the external open circuit, as displayed in Figure 5f.

**Figure 6:**
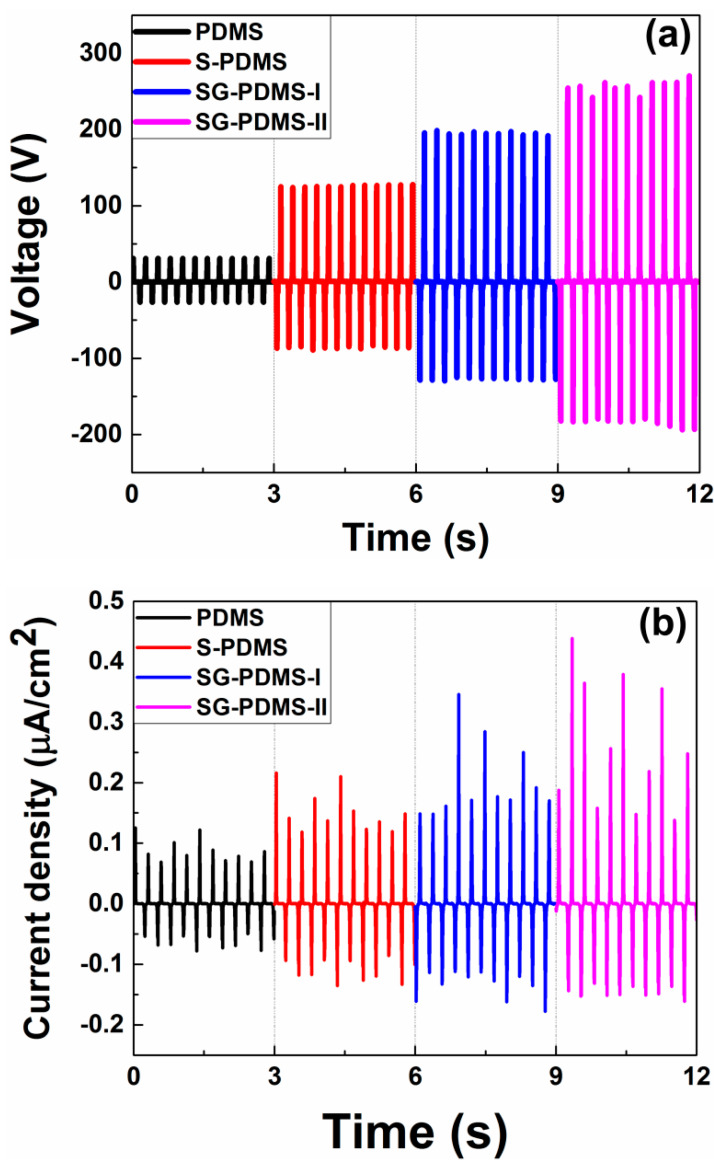
(**a**) Open-circuit output voltage and (**b**) short-circuit current density of TENG devices.

Open-circuit voltage output (V_OC_) performance of the TENGs was determined by applying an external pushing force. As shown in Figure 6a, the results clearly indicated that the V_OC_ of the TENGs was strongly dependent on surface roughness as well as GNP nanoparticle loading. The maximum value of the V_OC_ of pristine PDMS was 30.93 V. On the other hand, after Na_2_CO_3_ salt modification of S-PDMS, the V_OC_ increased to 95.48 V, which was about three times higher than that of pristine PDMS. This demonstrated that the increased surface area eventually improved the total amount of charge generated during contact. Adding GNP nanoparticles with the same Na_2_CO_3_ concentration offered V_OC_ values of 198.61 and 270.19 V for 0.03 wt.% (SG-PDMS I) and 0.50 wt.% (SG-PDMS II) GNP loading, respectively. These achieved output performances were approximately 6.4 and 8.7 times higher than that of pristine PDMS. This significant improvement of the V_OC_ was attributed to the increased surface charge density due to the coupled effect of the Na_2_CO_3_ template and GNP nanofillers. These results were also supported by the surface roughness (Figure 2c,d), SEM images (Figures S1 and S2) and charge density results (Figure S4).

The output current performance of the TENGs at fixed loading resistance of 10 MΩ was also investigated. The maximum output current density was observed as 0.127, 0.216, 0.346 and 0.438 μA/cm^2^ for the pristine PDMS, S-PDMS, SG-PDMS-I and SG-PDMS-II, respectively (Figure 6b). These values indicated that the output current exhibited a similar trend to output voltage (i.e., the coupled effect of the Na_2_CO_3_ template and GNP nanoparticles yielded a high output current performance due to the increased surface area and charge density).

### 3.3. Mechanical Durability of SG-PDMS-II@[PVA/GNP-PSS]_3_ TENG

**Figure 7:**
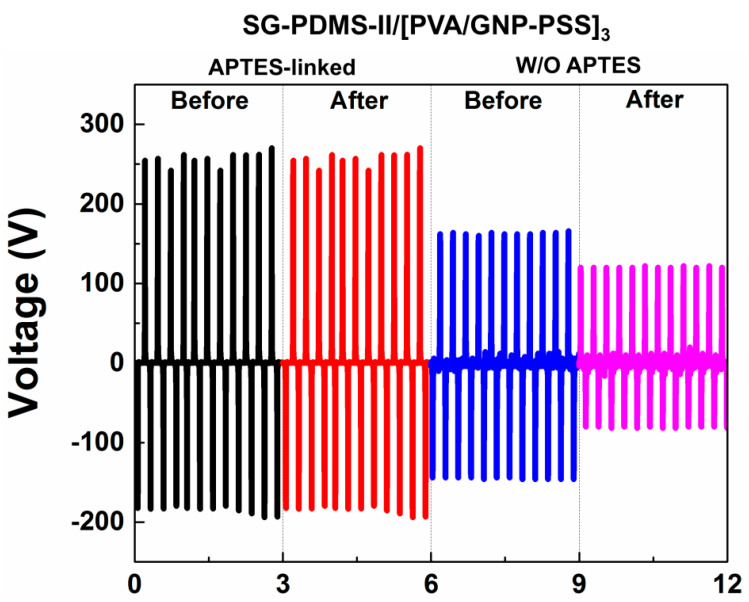
Triboelectric output voltage of SG-PDMS-II@[PVA/GNP-PSS]_3_ TENG with and without an APTES linker before and after 10,000 bending cycles.

In order to confirm the prediction that TENG performance is improved due to the chemical bonding between PDMS and the electrode by adding APTES, the V_OC_ of APTES-linked SG-PDMS-II TENG before and after 10,000 bending cycles was measured and compared to that of the control sample not linked by APTES. This was to investigate the effect of an APTES linker on mechanical stability and electrical output performance during repeated operating cycles. As shown in Figure 7, APTES-linked TENG delivered high and consistently similar output voltage after 10,000 bending cycles. On the other hand, the V_OC_ of non APTES-linked TENG was lower, and it decreased significantly after 10,000 bending cycles, confirming the poor mechanical durability. This was attributed to the weak adhesion of PSS and PDMS surfaces, while the enhanced V_OC_ for APTES-linked TENG was due to the reduced charge leakage between the PDMS-based TM and GNP electrode. These findings confirmed that the chemical bonding between the GNP-PDMS dielectric layer and the GNP electrode improved both the output performance and durability of the TENG.

The performance of modified PDMS with GNP and Na_2_CO_3_ in this work compared to other modified PDMS-based TENGs reported in the literature, such as Na_2_CO_3_-PDMS/TENG, obtained 125 V, which was five times higher than the pristine PDMS [15]. The V_OC_ was ~108 V and ~80 V for the TENG based on aligned graphene sheet/PDMS and GNP/PDMS, respectively, which was much higher than that of the pristine PDMS film; the V_OC_ increased with load resistance [21]. However, as shown in Figure 6a, this work showed better performance; V_OC_ was ~270.2 V due to coupling modifications and reducing charge loss between the electrode and modified PDMS.

To summarize, our work of coupling modification of PDMS-based TENG mainly had the following three achievements: (1) the improved capacitance of PDMS from oxygen functional moieties of GNP; (2) increased surface area of PDMS, with the use of Na_2_CO_3_ as a sacrificial template; (3) reduced charge leakage between the electrode and modified PDMS film, with the use of chemical bonding of modified GNP-PDMS and [PVA/GNP-PSS]_3_ through APTES. All of these achievements played a critical role for the higher energy harvesting performance of TENG.

## 4. Conclusions

A PDMS-based TENG was successfully fabricated for the generation of electrical energy. Coupled modification of pristine PDMS using a Na_2_CO_3_ template and GNP filler improves the output performance due to the enhanced effective contact area, compressive stress and surface charge density, which reached 54.49 μC/m^2^ for SG-PDMS-II/TENG and is 2.5 times higher than the pristine PDMS TENG. The modified PDMS also delivered an open-circuit voltage and short-circuit current density of up to 270.2 V and 0.44 μA/cm^2^, which are 8.7 and 3.5 times higher than those of the pristine PDMS, respectively. Moreover, chemical bonding between the modified PDMS layer and LbL-assembled PVA/GNP-PSS–stabilized graphene multilayer electrode significantly improved the output performance as well as the mechanical durability of the TENG. This simple and effective TENG fabrication process could be a useful approach for the development of other high performance TENGs.

## Supplementary Materials

The following are available online at https://www.mdpi.com/1996-1944/13/18/4156/s1, Figure S1: Top view SEM images of composite films. (a) PDMS; (b) S-PDMS; (c) SG-PDMS-I and (d) SG-PDMS-II, Figure S2: Cross-section view SEM images of composite films. (a) PDMS; (b) S-PDMS; (c) SG-PDMS-I and (d) SG-PDMS-II, Figure S3: Schematic illustration of composite films. (a) PDMS; (b) S-PDMS; (c) SG-PDMS-I and (d) SG-PDMS-II, Figure S4: Charge density of PDMS/TENG, SG-PDMS-I/TENG and SG-PDMS-II/TENG, Figure S5: A finite-element simulation of the potential distribution in the SG-PDMS-II/TENG using COMSOL Multiphysics® software. (a) fully contact phase; (b) separate phase; (c) fully separated phase and (d) contact phase.

## References

[1] Niu S., Wang Z.L. (2015). Theoretical systems of triboelectric nanogenerators. Nano Energy.

[2] Sun H., Tian H., Yang Y., Xie D., Zhang Y.-C., Liu X., Ma S., Zhao H.-M., Ren T.-L. (2013). A novel flexible nanogenerator made of ZnO nanoparticles and multiwall carbon nanotube. Nanoscale.

[3] Wang Z.L. (2014). Triboelectric nanogenerators as new energy technology and self-powered sensors—Principles, problems and perspectives. Faraday Discuss..

[4] Lee S., Yeom B., Kim Y., Cho J. (2019). Layer-by-layer assembly for ultrathin energy-harvesting films: Piezoelectric and triboelectric nanocomposite films. Nano Energy.

[5] Wang Z.L., Chen J., Lin L. (2015). Progress in triboelectric nanogenerators as a new energy technology and self-powered sensors. Energy Environ. Sci..

[6] Fan F.-R., Tian Z.-Q., Wang Z.L. (2012). Flexible triboelectric generator. Nano Energy.

[7] Fan Y.J., Meng X.S., Li H.Y., Kuang S.Y., Zhang L., Wu Y., Wang Z.L., Zhu G. (2016). Stretchable porous carbon nanotube-elastomer hybrid nanocomposite for harvesting mechanical energy. Adv. Mater..

[8] Kim M.-K., Kim M.-S., Kwon H.-B., Jo S.-E., Kim Y.-J. (2017). Wearable triboelectric nanogenerator using a plasma-etched PDMS-CNT composite for a physical activity sensor. RSC Adv..

[9] Zhao Z., Huang Q., Yan C., Liu Y., Zeng X., Wei X., Hu Y., Zheng Z. (2020). Machine-washable and breathable pressure sensors based on triboelectric nanogenerators enabled by textile technologies. Nano Energy.

[10] Kim D., Park S.-J., Jeon S.-B., Seol M.-L., Choi Y.-K. (2016). A triboelectric sponge fabricated from a cube sugar template by 3D soft lithography for superhydrophobicity and elasticity. Adv. Electron. Mater..

[11] Uddin A.I., Chung G.-S. (2017). Wide-ranging impact-competent self-powered active sensor using a stacked corrugated-core sandwich-structured robust triboelectric nanogenerator. Sens. Actuators B Chem..

[12] Xu Z., Duan J., Li W., Wu N., Pan Y., Lin S., Li J., Yuan F., Chen S., Huang L. (2019). Boosting the efficient energy output of electret nanogenerators by suppressing air breakdown under ambient conditions. ACS Appl. Mater. Interfaces.

[13] He X., Guo H., Yue X., Gao J., Xi Y., Hu C. (2015). Improving energy conversion efficiency for triboelectric nanogenerator with capacitor structure by maximizing surface charge density. Nanoscale.

[14] Sengupta D., Pei Y., Kottapalli A.G.P. (2019). Ultralightweight and 3D squeezable graphene-polydimethylsiloxane composite foams as piezoresistive sensors. ACS Appl. Mater. Interfaces.

[15] Cui C., Wang X., Yi Z., Yang B., Wang X., Chen X., Liu J.-Q., Yang C. (2018). Flexible single-electrode triboelectric nanogenerator and body moving sensor based on porous Na_2_CO_3_/polydimethylsiloxane film. ACS Appl. Mater. Interfaces.

[16] Chen J., Guo H., He X., Liu G., Xi Y., Shi H., Hu C. (2015). Enhancing performance of triboelectric nanogenerator by filling high dielectric nanoparticles into sponge PDMS film. ACS Appl. Mater. Interfaces.

[17] Jang S., Oh J.H. (2018). Rapid fabrication of microporous batio 3/pdms nanocomposites for triboelectric nanogenerators through one-step microwave irradiation. Sci. Rep..

[18] Seol M.-L., Woo J.-H., Lee D.-I., Im H., Hur J., Choi Y.-K. (2014). Nature-replicated nano-in-micro structures for triboelectric energy harvesting. Small.

[19] Park K.-I., Bin Bae S., Yang S.H., Lee H.I., Lee K., Lee S.J. (2014). Lead-free BaTiO_3_ nanowires-based flexible nanocomposite generator. Nanoscale.

[20] Song G., Kim Y., Yu S., Kim M.-O., Park S.-H., Cho S.M., Velusamy D.B., Cho S.H., Kim K.L., Kim J. (2015). Molecularly engineered surface triboelectric nanogenerator by self-assembled monolayers (METS). Chem. Mater..

[21] Xia X., Chen J., Liu G., Javed M.S., Wang X., Hu C. (2017). Aligning graphene sheets in PDMS for improving output performance of triboelectric nanogenerator. Carbon.

[22] Kim W.-G., Tcho I.-W., Kim D., Jeon S.-B., Park S.-J., Seol M.-L., Choi Y.-K. (2016). Performance-enhanced triboelectric nanogenerator using the glass transition of polystyrene. Nano Energy.

[23] Zhang X.-S., Han M.-D., Wang R., Meng B., Zhu F., Sun X.-M., Hu W., Wang W., Li Z.-H., Zhang H. (2014). High-performance triboelectric nanogenerator with enhanced energy density based on single-step fluorocarbon plasma treatment. Nano Energy.

[24] Wang S., Xie Y., Niu S., Lin L., Liu C., Zhou Y.S., Wang Z.L. (2014). Maximum surface charge density for triboelectric nanogenerators achieved by ionized-air injection: Methodology and theoretical understanding. Adv. Mater..

[25] Fan F.-R., Lin L., Zhu G., Wu W., Zhang R., Wang Z.L. (2012). Transparent triboelectric nanogenerators and self-powered pressure sensors based on micropatterned plastic films. Nano Lett..

[26] Chun J., Kim J.W., Jung W.-S., Kang C.-Y., Kim S., Wang Z.L., Baik J.M. (2015). Mesoporous pores impregnated with Au nanoparticles as effective dielectrics for enhancing triboelectric nanogenerator performance in harsh environments. Energy Environ. Sci..

[27] Cui N., Gu L., Lei Y., Liu J., Qin Y., Ma X.-H., Hao Y., Wang Z.L. (2016). Dynamic behavior of the triboelectric charges and structural optimization of the friction layer for a triboelectric nanogenerator. ACS Nano.

[28] Wu C., Kim T.W., Choi H.Y. (2017). Reduced graphene-oxide acting as electron-trapping sites in the friction layer for giant triboelectric enhancement. Nano Energy.

[29] Park H.-W., Huynh N.D., Kim W., Lee C., Nam Y., Lee S., Chung K.-B., Choi D. (2018). Electron blocking layer-based interfacial design for highly-enhanced triboelectric nanogenerators. Nano Energy.

[30] Chung I.J., Kim W., Jang W., Park H.-W., Sohn A., Chung K.B., Kim H.-T., Choi D., Park Y.T. (2018). Layer-by-layer assembled graphene multilayers on multidimensional surfaces for highly durable, scalable, and wearable triboelectric nanogenerators. J. Mater. Chem. A.

[31] Kim D., Lee S., Ko Y., Kwon C.H., Cho J. (2018). Layer-by-layer assembly-induced triboelectric nanogenerators with high and stable electric outputs in humid environments. Nano Energy.

[32] Huang L.-B., Xu W., Tian W., Han J.-C., Zhao C.-H., Wu H., Hao J. (2020). Ultrasonic-assisted ultrafast fabrication of polymer nanowires for high performance triboelectric nanogenerators. Nano Energy.

[33] Beal J.H., Bubendorfer A., Kemmitt T., Hoek I., Arnold W.M. (2012). A rapid, inexpensive surface treatment for enhanced functionality of polydimethylsiloxane microfluidic channels. Biomicrofluidics.

[34] Cha C., Antoniadou E., Lee M., Jeong J.H., Ahmed W.W., Saif M.T.A., Boppart S.A., Kong H. (2013). Tailoring hydrogel adhesion to polydimethylsiloxane substrates using polysaccharide glue. Angew. Chem. Int. Ed. Engl..

