# Supplementary material for: Enhanced Triboelectric Performance of Modified PDMS Nanocomposite Multilayered Nanogenerators
Source: Materials (Basel). 2020 Sep 18;13(18):4156. doi: 10.3390/ma13184156 (PMC7560417; doi:10.3390/ma13184156)
Supplement: Supplementary file 1 [file materials-13-04156-s001.pdf]

materials

Supplementary Materials

Enhanced Triboelectric Performance of Modified PDMS Nanocomposite Multilayered Nanogenerators

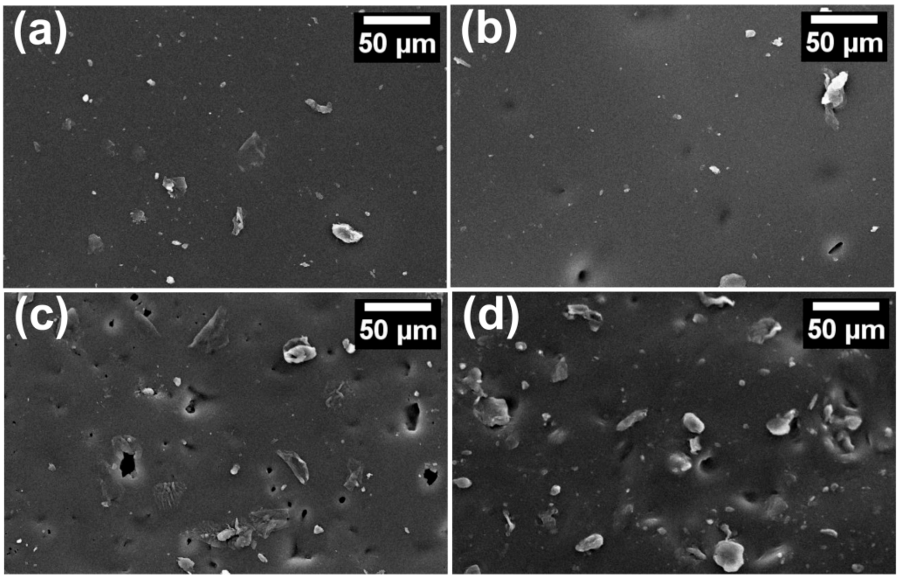

Figure S1. Top view SEM images of composite films. (**a**) PDMS; (**b**) S-PDMS; (**c**) SG-PDMS-I and (**d**) SG-PDMS-II.

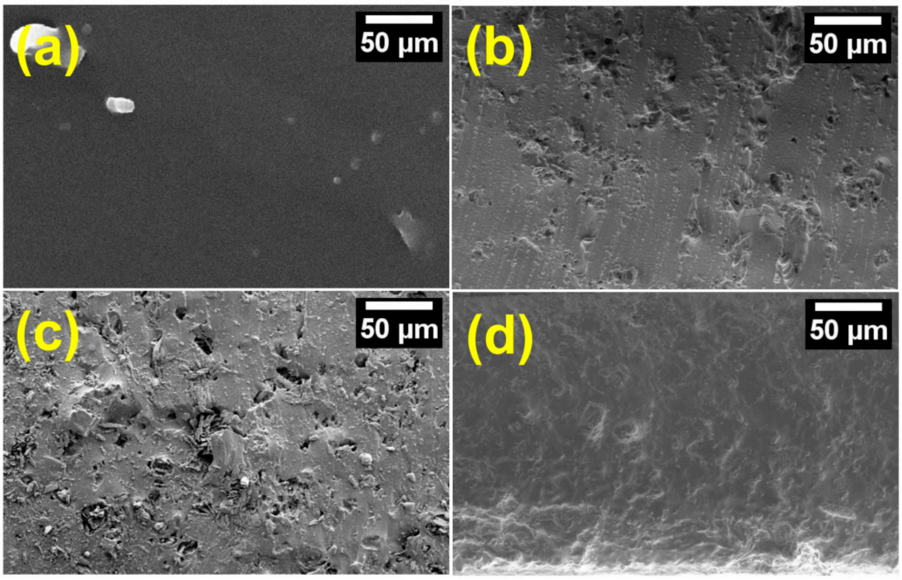

Figure S2. Cross-section view SEM images of composite films. (**a**) PDMS; (**b**) S-PDMS; (**c**) SG-PDMS-I and (**d**) SG-PDMS-II.

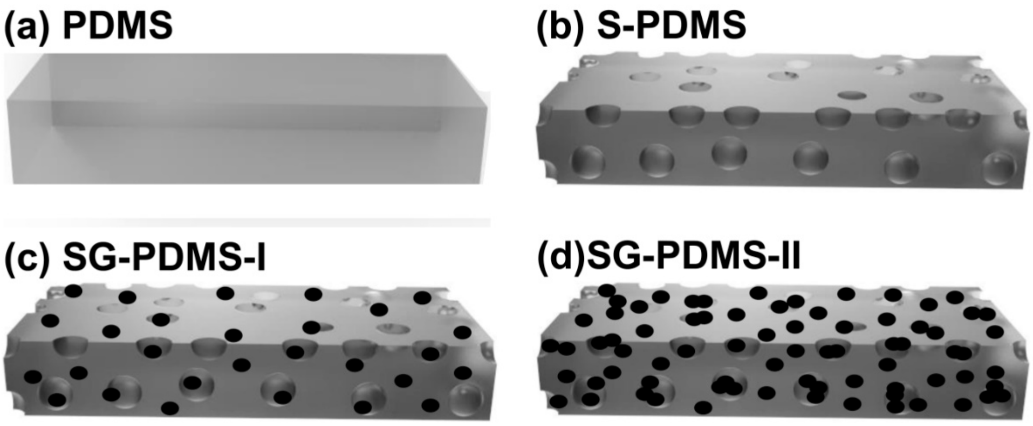

Figure S3. Schematic illustration of composite films. (**a**) PDMS; (**b**) S-PDMS; (**c**) SG-PDMS-I and (**d**) SG-PDMS-II.

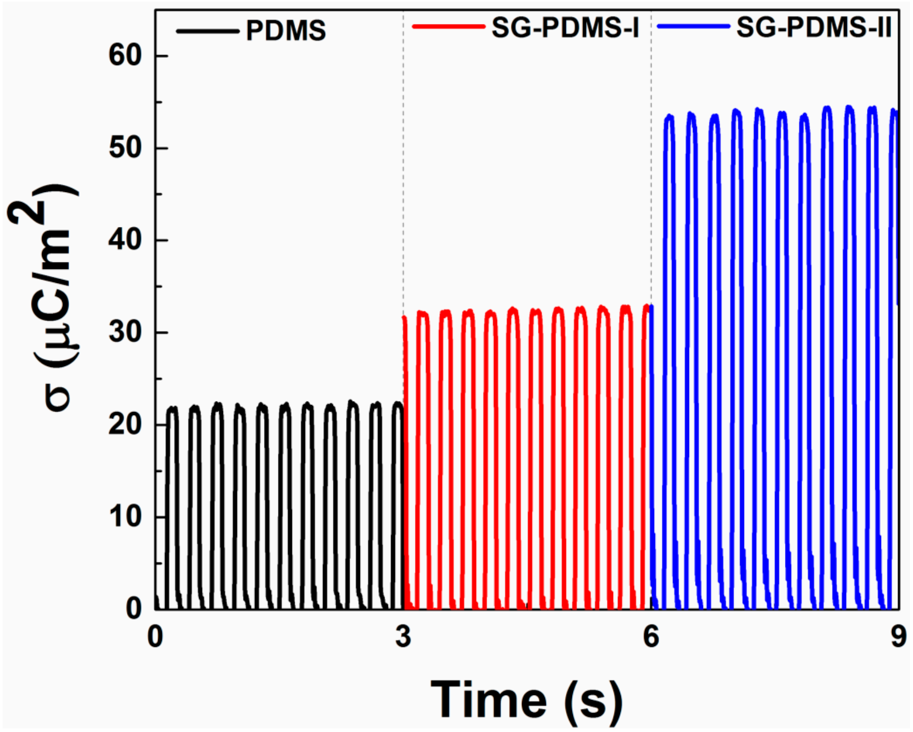

Figure S4. Charge density of PDMS/TENG, SG-PDMS-I/TENG and SG-PDMS-II/TENG.

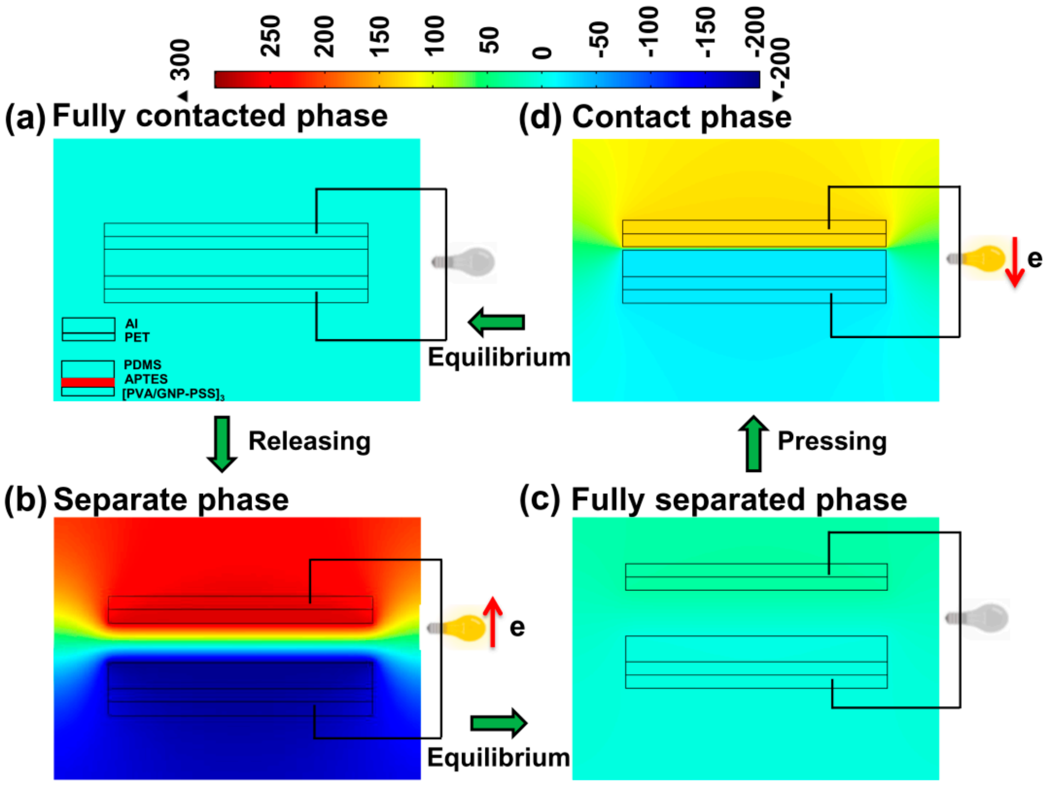

Figure S5. A finite-element simulation of the potential distribution in the SG-PDMS-II/TENG using COMSOL Multiphysics® software. (**a**) fully contact phase; (**b**) separate phase; (**c**) fully separated phase and (**d**) contact phase.

© 2020 by the authors. Submitted for possible open access publication under the terms and conditions of the Creative Commons Attribution (CC BY) license (http://creativecommons.org/licenses/by/4.0/).
